# Perceived Clinician Autonomy Support Before and After the COVID-19 Pandemic Onset in a Pregnancy Cohort

**DOI:** 10.1007/s10995-026-04286-9

**Published:** 2026-06-03

**Authors:** Mark Fedyk, Katherine B. Glaser, Andrea Millman, Saher Daredia, Charles P. Quesenberry, Assiamira Ferrara, Susan D. Brown

**Affiliations:** 1 Department of Internal Medicine, School of Medicine, University of California Davis Health, 4150 V St, Sacramento, CA 95817 USA; 2 Department of Obstetrics and Gynecology, College of Medicine, University of Arizona, Tucson, AZ USA; 3 Division of Research, Kaiser Permanente Northern California, Oakland, CA USA; 4 Kaiser Permanente Bernard J. Tyson School of Medicine, Pasadena, CA USA

**Keywords:** Patient autonomy, Patient-centered care, Patient-clinician relationship, COVID-19 pandemic.

## Abstract

**Background:**

Patient autonomy — the ability to form intentions and undertake actions based upon one’s values — is essential to patient-centered care. However, little research has examined patients’ perceptions of autonomy support, defined as clinician behaviors that help patients understand options, ask questions, feel heard, and make decisions aligned with their values. This is a critical gap, particularly for pregnant and postpartum populations.

**Methods:**

Our objective was to examine a measurable aspect of the patient-clinician relationship that could provide a window into perceived autonomy, specifically, whether patients reported clinician behaviors consistent with autonomy-supportive care. Measured using a subset of responses to the 6-item short-form of the validated Healthcare Climate Survey, we examined whether perceived autonomy support from clinicians shifted from pre- to post-onset of the COVID-19 pandemic and descriptively explored whether patterns appeared to vary by participant characteristics. We analyzed a diverse subset of participants in the Pregnancy, Lifestyle and Environment Study-2 (PETALS-2), a prospective longitudinal cohort study. Participants completed validated surveys at baseline during pregnancy (2017–2019, pre-pandemic onset) and at a study follow-up at 24–36 months postpartum (2020–2021, post-pandemic onset).

**Results:**

In the analytic sample (*N* = 278), perceived autonomy support decreased over time, both overall (baseline 95% CI = 5.3–5.8 and follow-up 95% CI = 4.8–5.3, *p* < 0.001). Descriptive subgroup analyses suggested similar directional patterns across several racial/ethnic groups, but subgroup results were interpreted cautiously because the study was not powered to test differences by race/ethnicity. These data therefore do not provide sufficient evidence to draw conclusions about whether changes in perceived autonomy support differed by sociodemographic characteristics or recent perceived discrimination.

## Significance

Perceived clinician autonomy support declined between pregnancy baseline and postpartum follow-up, a period spanning the onset of the COVID-19 pandemic. Because postpartum transition and pandemic-related health-system changes occurred simultaneously, these findings should be interpreted as exploratory.

## Introduction

Autonomy — the ability to form intentions based upon one’s authentic wants, desires, preferences, and goals and to be able to live in such a way that typically those intentions are not frustrated when put into action — is essential to many health protective or otherwise beneficial behaviors in patients(Brody, 1985). Autonomy matters in physical rehabilitation no less than it matters for pediatric family medicine or palliative care: it is hard to find examples of effective clinical care where autonomy is not important (Georgeff et al., 1999; O’Neill, 2002; Schmidt et al., 2012).

Clinicians interact with the autonomy of their patients by acting to support it. This simple idea is the essential core of patient-centered care (Ells et al., 2011; Frank, 2013; Lee & Lin, 2010), but it is also the mechanism by which the ethical ideal of autonomy is translated into practice. The quality and level of autonomy support that clinicians provide should therefore be perceptible to most patients(Brown et al., 2019), suggesting that it should be possible to track patient autonomy through self-reported instruments which measure perceived autonomy.

Determining whether it is possible to track patient autonomy matters because, in clinical practice, autonomy support overlaps substantially with shared decision-making (Deherder et al., 2022; Kassahun & Zewdie, 2022; Olwanda et al., 2024; Vedam et al., 2019). Shared decision-making occurs when clinicians provide information about medically reasonable options, elicit patient preferences and values, and support patients in deliberating about choices, and it is a well-studied area in clinical research (Arnold et al., 2008; Edwards & Elwyn, 2009; Epstein, 2013). Shared-decision making is especially relevant in pregnancy and postpartum care, where patients often face preference-sensitive decisions and where respectful communication may affect perceived trust, dignity, and agency. In contrast to paternalistic models of care (Sandman & Munthe, 2009, 2010), shared decision-making provides a concrete clinical mechanism through which autonomy support can be enacted.

The present study examines whether autonomy support can be measured in a population of pregnant and postpartum patients. Though this study does not assess morbidity or mortality in pregnant or postpartum patients, given the rate of such events in the United States as compared to other industrialized nations and the recognition of reproductive coercion and racism (Hoyert, 2024; Thoma & Declercq, 2023), patient autonomy in pregnancy has tremendous importance. We examined changes in patient autonomy support by analyzing responses to the validated Healthcare Climate Survey (Czajkowska et al., 2017; Schmidt et al., 2012; Williams et al., 2006). Because survey responses were collected before and after the onset of the COVID-19 pandemic, the study provided an opportunity to examine changes in perceived autonomy support across a period of major health-system disruption. We therefore analyzed our data with the goal of determining whether any significant change occurred in perceived autonomy support across a period that included the onset of the pandemic—which severely disrupted the provision of, and patients’ access to healthcare. Secondarily, because autonomy support is an important component of respectful and equitable care, we descriptively explored whether patterns of perceived autonomy support appeared to vary by sociodemographic characteristics or recent perceived discrimination; these analyses were exploratory and were not intended to provide definitive tests of subgroup differences.

## Methods

### Study Design and Sample

We analyzed data from the Pregnancy, Lifestyle and Environment Study-2 (PETALS-2), an ongoing prospective longitudinal cohort study among healthy individuals aged 18–45 years with a singleton pregnancy. Participants recruited from a large integrated health system in Northern California originally completed validated surveys at baseline (10–13 weeks of pregnancy) as part of the PETALS project (2017–2018; pre-pandemic onset)(Zhu et al., 2017). Subsequently, in PETALS-2, participants were invited to complete follow-up assessments with the last occurring at 24–36 months postpartum (July 2020 – October 2021; post-pandemic onset). The sample for this secondary data analysis consisted of 278 participants with available survey data on autonomy support at baseline and follow-up. All participants provided informed consent. The study was approved by the Kaiser Foundation Research Institute human subjects committee (IRB protocol no. 1295116). Study procedures were conducted in accordance with the ethical standards of the responsible institutional review board and with the 1964 Declaration of Helsinki and its later amendments, and we have followed the STROBE and SAMPL guidelines for reporting and interpretating the study data.

Because this was a secondary analysis restricted to participants with complete autonomy-support survey data at both baseline and follow-up, the analytic sample may differ from the larger PETALS/PETALS-2 cohort. We could not conduct a new comparison of included and excluded participants; therefore, potential selection into the analytic sample should be considered when interpreting this study’s results.

Race and ethnicity categories reflect those available in the PETALS-2 survey data; more specific Hispanic or Latina/o origin categories were not available for this analysis.

### Survey Components

Participant sociodemographic characteristics including age, race/ethnicity, education, household income, and parity were obtained from the baseline survey.

Autonomy support was assessed at baseline and follow-up using the 6-item short-form of the validated Healthcare Climate Survey (HCCS) (Czajkowska et al., 2017; Schmidt et al., 2012; Williams et al., 2006). HCCS items ask participants to characterize the impact that interactions with their physicians had on their autonomy (Table 1). Items are rated on a 7-point Likert-type scale from “strongly disagree” to “strongly agree.”

**Table 1:** .

Autonomy-Support Items from the Healthcare Climate Survey
I feel that my physician provides me choices and options.
I feel understood by my physician.
My physician conveys confidence in my ability to make changes.
My physician encourages me to ask questions.
My physician listens to how I would like to do things.
My physician tries to understand how I see things before suggesting a new way to do things.

Perceived autonomy scale scores were formed by averaging item responses and, in order to not impute a gradient to our data that would inappropriately influence our statistical analysis, rounding these averages to the nearest integer (Demirtas, 2009; Enders, 2022). We excluded participants missing data for any HCCS item (*n* = 21, i.e., 7.5% of the study sample).

Perceived discrimination was assessed at follow-up using items modified from the Commonwealth Fund 2001 Health Care Quality Survey (Princeton Survey Research Associates, 2002). Perceived discrimination in the prior 6 months is assessed using “yes/no” answers to the question stem, “I was judged unfairly or treated with disrespect because of my…” (race or ethnicity, gender, how well I speak English, or sexual orientation), as shown in Table 2. Since responses are “yes/no,” this allowed us to construct an index of perceived discrimination, consisting of the integers between 0 and 4, with 0 therefore indicating the absence of perceived discrimination.

**Table 2:** .

Perceived Discrimination Items^1^
I was judged unfairly or treated with disrespect because of my race or ethnic background.
I was judged unfairly or treated with disrespect because of how well I speak English.
I was judged unfairly or treated with disrespect because of my gender.
I was judged unfairly or treated with disrespect because of my sexual orientation.

^1^Items were adapted from the Commonwealth Fund 2001 Health Care Quality Survey

We therefore interpreted HCCS scores as patient-reported perceptions of clinician autonomy-supportive care, not as a direct measure of patients’ autonomous agency or objective clinician behavior.

### Analysis

We first examined whether perceived autonomy support changed from baseline to follow-up by comparing paired responses overall and descriptively within racial/ethnic groups. We also conducted exploratory analyses to assess whether perceived autonomy support appeared to vary by participant characteristics, including household income, educational attainment, age, and recent perceived discrimination. These analyses were treated as hypothesis-generating because the study was not designed or powered primarily to test subgroup differences, and because perceived discrimination was assessed only at follow-up. Accordingly, we did not interpret these analyses as definitive evidence for or against associations between participant characteristics and autonomy support (Gelman & Loken, 2013). Because these exploratory analyses did not materially alter interpretation of the primary within-participant change over time, we emphasize the primary longitudinal comparison and present subgroup and perceived-discrimination findings descriptively.

Statistical significance for the primary paired comparison was evaluated using a two-sided α = 0.01 to provide a conservative test of change over time.

## Results

Participant characteristics of the analytic sample are shown in Table 3. The sample was diverse, with over two-thirds of participants self-identifying with a racial or ethnic group other than white.

**Table 3:** .

*Participant characteristics of the analytic sample* (PETALS-2), *N* = 278	
	*n* (%) or Mean ± SD
Age at delivery (years)	
18–24	23 (8.3)
25–29	63 (22.7)
30–34	111 (39.9)
35–39	65 (23.4)
40–44	16 (5.8)
Race/ethnicity^1^	
Asian/Pacific Islander	66 (21.8)
Black/African American	31 (10.2)
Hispanic	99 (32.7)
Non-Hispanic white	118 (38.9)
Native American	13 (4.3)
More than one race/ethnicity	24 (7.9)
Education	
High school or less	32 (11.5)
Some college	66 (23.7)
College graduate	92 (33.1)
Postgraduate	88 (31.7)
Annual household income ($)^2^	
< 50,000	57 (20.7)
50,000–99,000	78 (28.3)
100,000–149,999	52 (18.8)
≥ 150,000	89 (32.2)
Parity	
0	141 (51.5)
1	95 (34.7)
2+	38 (13.9)
Gestational age at baseline assessment (weeks)	13.9 ± 2.4
Postpartum age at follow-up assessment (months)	29.6 ± 4.8

^1^The sum of these categories is greater than the sample size, as participants who identified as “more than one race/ethnicity” were included in each of the categories they identified with

^2^Missing data for annual household income (*n* = 2), gestational age at baseline assessment (*n* = 4), and parity (*n* = 4) lead to the exclusion of data from some participants

Scale scores for perceived autonomy ranged from an average of 5.56 (SD = 1.40) at baseline and an average of 5.12 (SD = 1.55) at follow-up. Analyses examining this change over time indicated that the decrease from baseline (95% CI = 5.3–5.8) to follow-up (95% CI = 4.8–5.3) was statistically significant (Paired t-test, *n* = 263, for α = 0.01; *p* < 0.001).

We also examined descriptive estimates by race/ethnicity to assess whether the direction of change appeared broadly similar across groups. Descriptive estimates by race/ethnicity showed lower follow-up HCCS scores than baseline scores in several groups (Table 4); however, as noted, these subgroup analyses were exploratory and were not designed to test whether changes differed significantly across racial/ethnic groups.

**Table 4:** .

Perceived autonomy support^1^ over time by race/ethnicity		
	Baseline HCCS score (95% α) CI	Follow-up HCCS score (95% α) CI
Race/ethnicity^2^		
Non-Hispanic white (*n* = 113)	5.5–5.9	4.7–5.2
Black/African American (*n* = 89)	5.0–5.6	4.6–5.2
Asian/Pacific Islander (*n* = 61)	4.7–5.4	4.6–5.3
Hispanic (*n* = 99)	5.3–5.9	4.8–5.5

^1^The sum of these categories is greater than the sample size, as participants who identified as “more than one race/ethnicity” were included in each of the categories they identified with

^2^Missing data for annual household income (*n* = 2), gestational age at baseline assessment (*n* = 4), and parity (*n* = 4) lead to the exclusion of data from some participants

Exploratory analyses of perceived autonomy support by participant characteristics did not reveal clear or consistent patterns that would support strong conclusions about differences by household income, educational attainment, or age. Because these analyses were secondary and subgroup power was limited, we interpreted them cautiously and focused inference on the primary within-participant change over time.

**Fig. 1:**
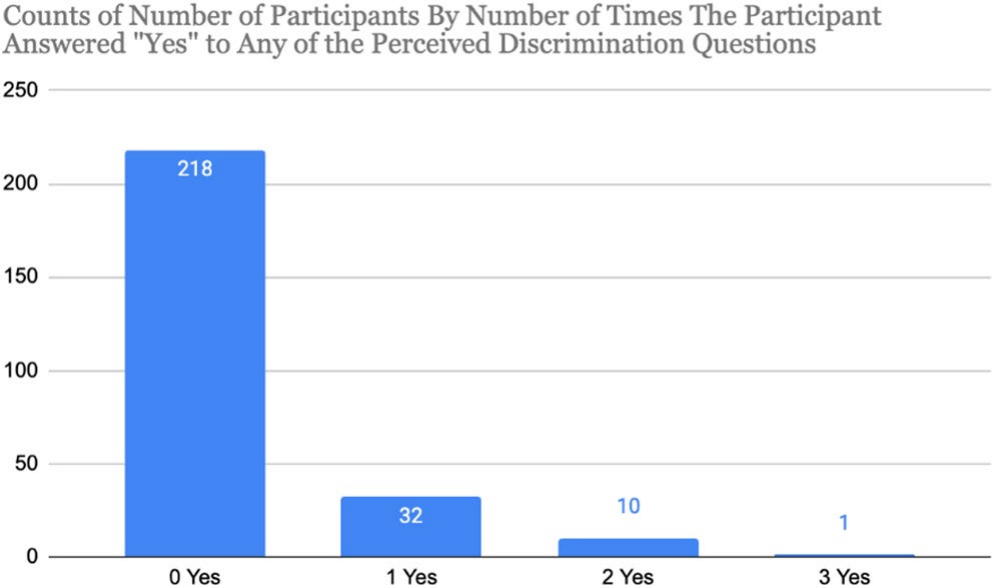
.

Roughly 20% of respondents scored 1 or greater on the measure of recent perceived discrimination, indicating that approximately 1 in 5 reported being judged unfairly or treated with disrespect because of at least one measured identity-related characteristic in the prior 6 months. Because perceived discrimination was assessed only at follow-up and was not limited specifically to physician interactions, these data are best interpreted descriptively rather than as a definitive test of the relationship between discrimination and perceived clinician autonomy support. The distribution of perceived discrimination scores is shown in Fig. 1, which we provide to characterize the frequency of recent perceived discrimination in the analytic sample, not to imply a tested association with autonomy support.

## Discussion

The present study leveraged longitudinal data to examine changes in patients’ perceived autonomy support from their healthcare providers over time, from before to after onset of the COVID-19 pandemic. Results indicate that perceived autonomy support decreased overall from pregnancy baseline to postpartum follow-up. Descriptive subgroup estimates suggested lower follow-up scores in several racial/ethnic groups, but these subgroup analyses were exploratory and were not designed to test whether changes differed significantly across groups. Exploratory analyses did not therefore provide a sufficient basis for drawing firm conclusions about whether autonomy support differed by sociodemographic characteristics or recent perceived discrimination. We therefore interpret the principal contribution of the study as evidence of an overall within-participant decline in perceived clinician autonomy support over time.

These findings should be interpreted in relation to prior work on patient-centered care, shared decision-making, and autonomy-supportive communication (Ronald M. Epstein & Street, 2011; Joosten et al., 2011; Sandman & Munthe, 2010). The HCCS items used in this analysis — including whether the physician provides choices and options, encourages questions, listens to patient preferences, and seeks to understand the patient’s perspective — overlap with core features of shared decision-making and patient-centered communication (Resnicow et al., 2022; Sandman & Munthe, 2009). The observed decline may therefore reflect changes not only in formal access to care, but also in the relational and communicative conditions under which participants experienced care during the pandemic and postpartum period. At the same time, because the present study did not measure visit modality, visit frequency, continuity of care, or specific decision-making episodes, it cannot determine which aspects of the patient-clinician relationship changed.

While the study design limits causal inference, these results are consistent with the possibility that pandemic-era health-system disruption contributed to the observed decrease. It is possible that the changes observed here reflect the massive disruptions in healthcare access wrought by the pandemic, the implications of which are yet unclear. Our survey data do not allow us to test any hypothesis about the mechanisms by which the pandemic may have influenced perceived autonomy support, and neither do we have data that allows us to begin to analyze the behavioral changes in patients which may or may not be causally linked with significant changes in perceived autonomy support. These are both important areas to focus on in further research.

To that end, an important alternative explanation of our results is the transition from pregnancy to postpartum care. Baseline surveys were completed during pregnancy, whereas follow-up surveys were completed 24–36 months postpartum. Participants’ care needs, visit frequency, clinician continuity, expectations of care, and salience of pregnancy-related decision-making may therefore have differed substantially across measurement occasions. The observed decline in autonomy support should therefore not be attributed solely to the COVID-19 pandemic. Further research can clarify this issue.

Future research should address the question of whether similar decreases in perceived autonomy can be found in different patient populations, beyond the pregnancy and postpartum cohort examined here. Analyses based in comparing differing populations may generate important insights about which patient populations benefit most from autonomy supportive interventions.

We did not draw firm conclusions about the relationship between recent perceived discrimination and perceived clinician autonomy support. One reason for caution is that the discrimination items asked whether participants had been judged unfairly or treated with disrespect in the prior 6 months because of identity-related characteristics, whereas the autonomy-support items asked specifically about interactions with the participant’s physician. These measures may therefore capture related but distinct experiences of care. For example, unfair treatment reported in the discrimination items may have occurred in settings or interactions other than encounters with the participant’s physician.

At a more general level, our results also indicate that there may be value in exploring how other ethical principles may be operationalized in the care of patients – especially patients who experience vulnerability. It should be possible to discover whether principles like those expressing ideals of fairness, beneficence, value-congruent care, and so on, are associated with improvements in patient satisfaction or health outcomes. Developing self-reported survey items that can probe clinical operationalizations of these principles would provide rich empirical guidance about where best to focus efforts to improve the ethics of healthcare.

As noted above, the study design precludes causal determinations about the cause of changes in perceived autonomy. Other limitations include the low representation of participants with lower levels of education, and the setting in a single healthcare delivery system. The study was also not designed primarily to test subgroup differences by race/ethnicity, socioeconomic position, or perceived discrimination. As a result, analyses involving these characteristics should be interpreted as exploratory rather than as evidence of the absence of inequities in autonomy-supportive care. A further limitation is that the analytic sample was restricted to participants with complete HCCS data at both time points. We did not compare these participants with excluded PETALS/PETALS-2 participants in the present revision, so selection into the analytic sample may limit generalizability.

Study strengths include the use of validated self-report measures to examine health care climate; the racially and ethnically diverse sample; and analyses that included not only standard sociodemographic factors, but perceived discrimination as an important dimension of social equity.

## Conclusion

In a diverse pregnancy/postpartum cohort, perceived clinician autonomy support declined between pregnancy baseline and 24–36 months postpartum follow-up, a period spanning the onset of the COVID-19 pandemic. The study cannot isolate the pandemic from postpartum transition, care setting changes, telehealth, or broader health-system disruption, but the observed decline warrants further investigation.
